# Financial immunity of companies from Indonesian and Shanghai stock exchange during the US-China trade war

**DOI:** 10.1016/j.heliyon.2022.e08832

**Published:** 2022-01-25

**Authors:** Liliana Inggrit Wijaya, Zunairoh Zunairoh, Rizky Eriandani, I Made Narsa

**Affiliations:** a Management Department, Faculty of Business and Economics, University of Surabaya, Surabaya, Indonesia; b Accounting Department, Faculty of Business and Economics, University of Surabaya, Surabaya, Indonesia; c Accounting Department, Faculty of Economics and Business, Airlangga University, Surabaya, Indonesia

**Keywords:** Financial immunity, Financial distress, Stock returns, The US-China trade war, Multi-country, Multidimensional

## Abstract

This study aims to examine the country-level financial immunity and vulnerability due to the multidimensional impact of the US-China trade war by using potential driving factors, namely financial and market performance, economic conditions, and government interventions. This study uses financial distress as a moderating variable to examine the relationship between financial immunity and stock returns of companies listed on the Indonesia Stock Exchange and the Shanghai Stock Exchange, which have different characteristics. The research samples were companies listed on the Indonesia stock exchange and the Shanghai stock exchange with 767 and 736 observations, respectively, in 2016–2019. The first-stage test method uses balanced data panel regression to test individual interactions as a sufficient condition. Regression model specification of the response variable for the fixed effects model can overcome the common effects model limitations. The second stage is a matched test or Paired Samples T-Test for hypothesis testing after testing each individual interaction for each country. The results of this study show that financial immunity has a positive effect on stock returns in two countries: Indonesia and China. Meanwhile, the financial distress of the US-China Trade War for Indonesia and Shanghai shows different results. Financial distress significantly reduces stock returns on the Indonesian Stock Exchange, while the distress does not affect stock returns on the Shanghai Stock Exchange. During the US-China trade war, trade policy uncertainty has caused economic policy uncertainty, thus triggering systemic risks in ASEAN markets, including Indonesia.

## 1 Introduction

The US-China trade war is the biggest challenge for the global economy because this trade war involves the world's two economic giants. The war began with the actions of former US President Donald Trump that imposed a 30–50% tariff on solar panel imports on January 22, 2018 (Costa and Sukartha, 2020). The situation got worse when the US imposed an additional 25% tariff on imported steel and an additional 10% tariff on imported aluminum for most major countries, including China (Wu and Turvey, 2021). On March 22, 2018, China responded by imposing additional tariffs of up to 25% on 128 US products (WTO, 2020). Comparing shipments in 2016–2017 to 2018–2019, US exports to China dropped by 15–27%, while US imports from China dropped by 14–23% (C. He et al., 2021). Since then, the two countries have retaliated against each other to raise tariffs for various goods imported from each other. As a result, there have been severe disruptions to the aggregate trade flows worldwide, such as crisis and business bankruptcy. While affected exporting firms have to cope with an increase in their product selling price, importers need to pay more for their purchases or find new suppliers. These things erode firms' competitiveness and reduce sales and profits, thus forcing them to form corporate immunity to withstand the crisis. To investigate the country-level financial immunity to the US-China trade war, this study raises three major categories: financial, economic stability, and government interventions variables (Ding et al., 2021; Zaremba et al., 2021).

The present study aims to examine the country-level financial immunity and vulnerability to stock returns due to the US-China trade war. The research design used is to collect all the potential driving factors multidimensionally, namely financial and market performance, economic conditions, and government interventions due to the US-China trade war. Previous studies on the trade war tend to focus on aspects of who wins and who loses the war (An et al., 2020; Nugroho et al., 2021) and the impact of the war on poverty, bankruptcy, and crises (Cui and Li, 2021; Shi et al., 2021; Wu and Turvey, 2021; Xu and Lien, 2020). The impact of country-level financial immunity on the US-China trade war has never been studied, especially in developing countries like Indonesia. This study uses a multi-country analysis where the results of this study show that financial immunity to overcome the US-China trade war varies between countries. This study focuses on financial immunity in Indonesia and China (Shanghai). The reason for choosing China is that in global competition, China is considered one of the Asian countries that has been significantly progressing, and what makes it even more interesting is that the US administration considers China's trade practices to be unfair in bilateral trade (Adekola, 2019; Lai, 2019). China's export value beats the US, making China the second-largest exporter of goods globally. This makes the US deficit larger because the US only exports a quarter of China's exports (Steinbock, 2018). Meanwhile, the reason for choosing Indonesia is that the US-China trade war negatively affects Indonesia because the two countries are Indonesia's largest export destination countries (BPS, 2020). The trade war makes Indonesia experience a significant economic impact, where Indonesia's trade balance is still in a deficit against China. When seeing Indonesia's non-oil and gas export market data, China ranks first while the US ranks third after Japan. Meanwhile, Indonesia is not overly dependent on imports from the US because Indonesia's largest non-oil and gas import market is China (Wulandari and Inayah, 2021).

This study contributes to the existing literature as follows. First, this study provides an analysis in overcoming the impact of the US-China trade war, namely the issue of financial immunity. Additionally, this is the first scholarly paper to analyze the vulnerability aspect to the crisis as an aspect that strengthens (or weakens) financial immunity in the US-China trade war as there has been no research analyzing this so far. Second, this study uses two contrasting points of view: the largest exporting country to Indonesia (China) and the developing country with the highest import value from China (Indonesia). Both points of view are very relevant to be analyzed as this study investigates financial immunity during the US-China trade war. Third, related to the methodological aspect, this study integrates financial immunity, trade war settlement, and multi-country analysis. This study uses an agnostic approach undertaking a series of methods arranged in several stages. The first stage is a series of panel data regressions that examine individual interactions. The next stage is to test dimensions for the financial and market, country characteristics, and policy responses aspects. However, Zaremba et al. (2021) postulate that a multidimensional information set has a weakness because it may potentially feature overlapping information contents and makes it irrelevant to use for decision making. Therefore, to overcome the overlapping information contents, this study applies a new approach with the features or dimensions of financial immunity, which are broken down into three major aspects, namely accounting and market financial, state economic conditions, and government interventions which will later be analyzed using the matched test or Paired Samples T-Test for hypothesis testing after testing each individual interaction for each country.

The remainder of the paper is organized as follows. Section 2 reviews the extant literature and develops some hypotheses of this research. Section 3 describes the construction of the sample and research model as well as statistical testing. Sections 4 and 5 outline the empirical strategies and discuss the research results. Finally, Section 6 presents conclusions, suggestions, and implications.

## 2 Literature review and theoretical basis

### 2.1 The US-China trade war

Interactions among nations have become very active globally, especially in international trade transactions. This condition triggers a high competition level to reach the best market from each country. The US-China trade war can become very damaging to the global economy. The war between the two countries disrupts the stabilization of international trade policies, which impacts not only on the two countries but also on other countries, including their trading partners. In the Shanghai Stock Exchange, there was a sharp decline in the index in early 2018 until the end of 2019, but after that, the Shanghai Stock Exchange condition gradually began to recover as indicated by an increase in the index to almost the same point as before the trade war (Hu and Wang, 2018). For LQ-45, the decline also occurred in 2018–2019 and began to recover after the trade war ended, although the conditions were not as good as before the trade war (Hu and Wang, 2018).

The theory of globalization is based on the problem of the global economy's structure that determines the processes of social development in every sector of the economy (An et al., 2020). International trade becomes an essential reference in encouraging economic development in a country. Every trade policy, both domestic and foreign, has the same goal: to increase state revenue in order to improve the nation's welfare.

**Figure 1:**
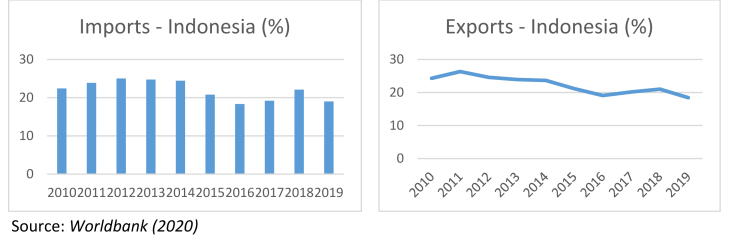
Indonesia's imports and exports in 2010–2019. Source: Worldbank, 2020.

From the economic aspect, a trade war is an economic conflict manifested by imposing restrictions on imports and exports between countries, such as import tariffs on goods, import bans on certain goods, and setting high standards on imported goods. The main determinant of the trade war was the high US trade deficit contributed by China, followed by the action of imposing each country's respective import-export policies (Nugroho et al., 2021; Shi et al., 2021). This trade war had a negative impact on the Chinese economy; retail sales in July 2019 decreased to 7.6%, lower than expectation of 8.6%, while the unemployment rate increased to 5.3% compared to June, which was only 5.1 % (Cui and Li, 2021; Nugroho et al., 2021; Shi et al., 2021; Xu and Lien, 2020). It is undeniable that the US-China are two countries with the largest economies in the world, so the dispute between the two affects the economic conditions of other countries, especially their main trading partner countries. Companies in Indonesia participate in increasing their trade through import and export activities undertaken in several countries, including the US and China. Figure 1 exhibits the declining trend of Indonesia's imports and exports in 2010–2019. This signifies that the trade war has increased global uncertainty and decreased investor optimism about the economy's future. Import and export activities themselves imply a country's economic conditions, which are closely related to the economic conditions of its trading partner countries. On the other hand, changes in the economy of trading partners will have a direct or indirect effect on the country's economic conditions.

### 2.2 Determinants of corporate financial immunity

This study considers various categories of variables that can boost the stock markets' immunity to the US-China trade war. The categories adopt Zaremba et al. (2021) and Ding et al. (2021), which include financial and market performance, economic conditions, and government interventions. The first category relates to companies' financial condition in Indonesia and China and their ability to cope with extreme market conditions. The companies’ ability to access internal and external funding can help to cope with the negative impact of the US-China trade war.

A declining stock market condition (bearish market) caused by the trade war can be overcome with a healthy balance sheet and financial position (An et al., 2020; Prager et al., 2018; Takahashi and Yamada, 2021). Policies taken under these conditions play a significant role in companies’ survival goals. An example of such policies is conservative investment policies. Companies need debt to expand aggressively. In a business cycle recession, when companies operate below their full capacity, revenues decrease, but expenses, particularly arising from paying debt obligations, do not tend to change significantly. The ensuing decrease in corporate earnings translates into a less immune stock market. The impact of the US-China trade war will be low if companies apply conservative investment policies to survive (Zaremba et al., 2021). The determinants of corporate financial immunity are explained as follows:

#### 2.2.1 Financial data

Financial data from the financial and market aspects are retrieved from companies' financial statements (Ding et al., 2021; Zaremba et al., 2021). Financial data is a company's financial position as the main determinant of corporate financial immunity to the US-China trade war. This study uses important financial ratio to survive, namely, return on assets. Furthermore, this study also uses indicators that were identified in country-level asset pricing studies, namely book-to-market value and price-earnings-ratio. The design of this study combines two variables that represent the stock market structure in two different countries: Indonesia and China. The following is an explanation of each ratio:

##### 2.2.1.1 Return on assets

This ratio is used to assess a company's financial condition from the accounting aspect. This ratio illustrates how much return is generated from the effective and efficient use of assets. The higher the ratio, the better the financial condition because the company can produce high returns in undertaking all forms of resource management. The formula for calculating ROA is as follows:

(1) $$\mathrm{Return}\;\mathrm{on}\;\mathrm{Assest}s_{i,t}=\frac{Earning\;after\;tax_{i,t}}{Total\;assets_{i,t}}$$

##### 2.2.1.2 Book-to-market value

This ratio is used to assess a company's financial condition from the market aspect. This ratio is calculated by dividing the book value by the market value. A high book to market value indicates that investors are less interested in the company's stocks, causing the stocks to be undervalued. The formula for calculating book to market value is as follows:

(2) $$\;Book\;to\;Market\;Value_{i,t}=\frac{Book\;value_{i,t}}{Market\;value_{i,t}}$$

##### 2.2.1.3 Price-earnings-Ratio

This ratio is used to measure the market value of a stock relative to its earnings. It is calculated by dividing the current stock price by the current earnings per stock. The ratio reflects the price of a stock experiencing undervalued or overvalued. Overvalued company stocks will reduce investor interest in investing and vice versa. The formula for calculating price earnings ratio is as follows:

(3) $$Price\;Earnings\;Ratio_{i,t}=\frac{Closing\;price_{i,t}}{Earning\;per\;share_{i,t}}$$

#### 2.2.2 Economic

In order to survive in times of crisis, a country's economic performance and environment play a critical role in dealing with a trade war.

**Figure 2:**
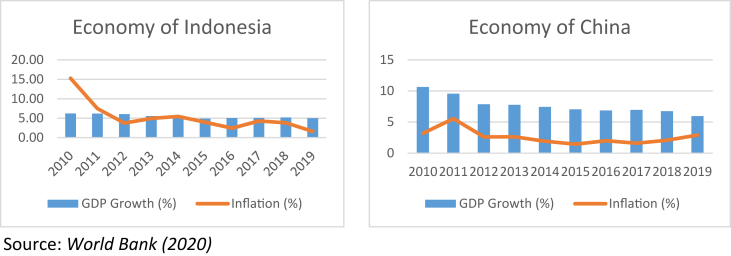
Economic conditions of Indonesia and China. Source: Worldbank, 2020.

The US-China trade war is an economic conflict between the two countries that can affect many countries' macroeconomic performance (C. He et al., 2021; Kida, 2020; Shi et al., 2021; Xu and Lien, 2020). This study includes several macroeconomic variables that cast light on a country's economic growth, such as gross domestic product and inflation. Gross domestic product (GDP) is the total production of goods and services in a country produced using domestic resources, while inflation is a criterion for the price of domestic goods to increase (Inaba, 2020; Thampanya et al., 2020). A high inflation rate will reduce the actual income earned by investors. Figure 2 exhibits the economic conditions of Indonesia and China based on the annual GDP growth and inflation. According to the Worldbank (2020), China had an annual GDP growth of 10.6% (2010) and a downward trend to 6% (2019). Meanwhile, Indonesia's GDP growth has never been above 6% since 2010. This difference can also be seen from the inflation condition in Indonesia, which had a downward trend of 1.6% (2019) and China had an upward trend of 2.9% since 2017 (2019). These changes are indicated due to the US-China trade war.

#### 2.2.3 Government interventions

The role of government in economic development is critical to a more prosperous society. The role is indispensable in overcoming market failures such as price rigidity, monopolies, and externalities that are detrimental to the state. This role can be carried out through direct or indirect interventions. Government interventions refer to a government's intentional interference to influence a country's resource allocation and market mechanisms. The interventions are divided into several categories, namely regulations, subsidies, monetary, fiscal policies, and accountability to society. The role of government interventions is a common occurrence in economic development (Zaremba et al., 2021). Government interventions should be carried out to overcome a country's problems such as unemployment and limited employment opportunities, poverty (Ding et al., 2021), income distribution, inflation (McKibbin, 2020), poor corporate governance (CG), and low social responsibility awareness.

The GCG factor is an approach that promotes an information transparency system regarding the management of go-public companies that focuses on stakeholder theory. In recent years, one of the important GCG indicators for monitoring focuses on the economic implications of corporate social responsibility (CSR). More than 80% of multinational companies have incorporated CSR as a global management strategy to improve the level of information transparency (Durand and Vergne, 2014; Kong et al., 2020). Investor confidence can be increased by environmental disclosure as the axis of CSR to identify risks sustainably.

CSR is very beneficial in global phenomena that occur in various countries. The implementation of social responsibility in Indonesia is not only regulated by the central government but also local governments, both provincial and district or city. Apart from being a company strategy in gaining customer trust (Feng et al., 2020; Singh and Misra, 2021), CSR has direct and indirect benefits and impacts for stakeholders. For the community, CSR can prevent corruption (Joseph et al., 2016). For companies, CSR can reduce risk and improve performance (Hsu and Chen, 2021). While CSR is categorized into two implementation patterns: activities carried out simultaneously with the community whose benefits can be directly felt by the community and activities carried out disjointly with the community, but the benefits can be felt directly by the community. In their operations, companies cannot be separated from the environment. If the CSR program is regarded as one of the strategic aspects in operations, its implementation is included in the operational costs. If not managed properly, these costs will negatively impact the companies. Companies must consider various stakeholders so that CSR can help reduce the risk of losing one or more support from stakeholders (Giuseppina, 2021). In this study, government interventions are measured by implementing each company's corporate social responsibility (CSR) in each country. This variable uses a dummy variable, namely 1 if the company implements corporate social responsibility and 0 if not.

### 2.3 Financial distress

Financial distress is a condition in which a company cannot meet its current obligations. Financial distress can enforce companies from two aspects, namely direct (legal, administrative, and consulting costs related to bankruptcy) and indirect (decreased business operations, lower employee morale, etc.). Companies facing high liquidation costs tend to choose capital structures in such a way that financial distress becomes less likely (Bose et al., 2021).

The measurement of this financial distress variable adopts the Zmijewski model. The reason for choosing the Zmijewski model is because this model has proven to be effective and quite accurate in predicting a firm's financial health over the last twenty decades (Qiu et al., 2020). Besides, the model can also predict corporate failure (Almamy et al., 2016). This model assesses how much financial distress a firm has by combining the three main pillars of the firm's financial ratios, namely liquidity, profitability, and solvency. The liquidity ratio is a ratio that can be used to measure firms' ability to meet their short-term obligations on time (Andreou et al., 2021). Firms will be considered good if they have sufficient liquidity to finance their operations and obligations in a timely manner. The profitability ratio indicates the condition of firms' performance in terms of profitability to achieve their economic position. Finally, solvency is firms' ability to finance their assets with debt. These three ratios are used in this model. The Zmijewski method's assessment criteria are that if Z < 0.5, the firm is declared healthy and vice versa. The formula used is as follows (Saputri et al., 2021):

(4) $$Z=-4.3-4.5X_{1}+5.7X_{2}+0.004X_{3}$$

Where, X_1_ is return on assets, X_2_ is total debt to total assets, X_3_ is current assets to current liability.

The Zmijewski model has a cutoff value of 0, with the assessment criteria:

1. If a firm's score is less than 0 (X < 0), then the firm is in non-financial distress (healthy).
2. If a firm's score is more than 0 (X > 0), then the firm is predicted to experience financial distress.

According to Michalkova et al. (2018), some of the causes of financial distress are as follows:

1. Neoclassical model: financial distress occurs due to an error in unraveling information contained in the financial statements, while the information is used as a basis for resource allocation.
2. Financial model: financial distress is characterized by the wrong financial structure that causes liquidity constraints so that the firm will not last long.
3. Corporate governance model: financial distress occurs due to a poor corporate governance system; thus, even though the firm has the right asset composition and good financial structure, it cannot create corporate sustainability.

### 2.4 Financial immunity and stock return

Financial immunity refers to a company's performance from financial and market, economic conditions, and government interventions aspects. The company's performance from the financial and market aspects reflects its ability to manage assets and debts, both short- and long-term, and make a profit. In asset management, companies must have optimal managerial capabilities to avoid risks. To be sustainable, companies need to have competent managers in asset management (Ding et al., 2021; Grundke and Moser, 2019; C. He et al., 2021). Competent managers will affect companies' financial condition by making a profit, reducing costs, and optimizing asset management. In managing debt, both short- and long-term, the most significant contribution is to reduce the cost of capital and maintain liquidity, which is reflected in companies' ability to pay both short- and long-term obligations. In this condition, companies will perform a financing mix by using external funds to finance the companies in addition to internal funds from retained earnings. If the proportion of debt exceeds companies' ability to pay their obligations, they will experience financial risk. Investors are more interested in buying stocks of companies that can make high profits, have optimal debt management, and have competitive stock prices. High investor demand for company stocks makes the stocks more attractive; thereby, stock returns are expected to be higher.

In addition to companies' performance from the financial and market aspects, financial immunity can also be seen from the aspect of the economic conditions. Gross Domestic Product (GDP) and inflation are references in seeing the condition of a nation. Government interventions that help build country-level financial immunity would subsequently benefit investors (Cui and Li, 2021; Wu and Turvey, 2021; Zaremba et al., 2021). In this case, CSR is considered a long-term company investment. Therefore, CSR can also be used as a corporate strategy in forming a positive brand image as a company that cares about the environment. Socially responsible companies are considered as companies that not only run their own business but also care about the surrounding environment, which subsequently attract investors (Ajina, 2019; Alsaadi et al., 2017; Boubaker et al., 2020). Good company performance, combined with favorable country conditions and government interventions, can attract investors to invest in that country. This indicates that the company has strong financial immunity. Companies with financial immunity will be more immune to corporate risks. As a result, investors are interested in investing because companies' prospects are good so that they can get high returns. The number of investors interested in investing increases companies’ stock prices and value.

The US-China trade war has made the condition of several countries unstable both from company and market performance and the countries' economic conditions aspects (Ding et al., 2021; Zaremba et al., 2021). Direct impact occurs mainly in countries directly involved in the trade war, namely the US and China. Conditions before and during the trade war are different because some cash flows should have entered but are hampered or even stopped. In consequence, the financial immunity of companies in the countries also decreases. In such conditions, investors will re-select their investment. Companies that are significantly affected will be reflected in their low level of financial immunity. As a result, there is a tendency that these stock investments will be less attractive to investors, thus decreasing stock prices and stock returns.

H1 Financial immunity has a positive effect on stock returns.

H2 Financial immunity will be lower during the US-China trade war.

H3 Stock returns will be lower during the US-China trade war.

### 2.5 Financial immunity, financial distress, and stock return

As time goes by, companies must continue running their business and dealing with fierce competition. In this condition, companies must provide the best service and have competitive prices in meeting consumer demand (Almamy et al., 2016; Andreou et al., 2021). If companies cannot meet consumer demand, they will go bankrupt sooner or later because there is no cash inflow. As a result, they are experiencing financial difficulties reflected by their difficulties paying their obligations to creditors or suppliers of funds. Companies with strong financial immunity but high financial distress would have lower immunity. Financial distress will also give a signal to investors that companies are experiencing high financial risk. The risk that investors do not tolerate makes the companies unattractive (C. He et al., 2021). Consequently, the companies' stock prices will decrease. During the US-China trade war, companies’ performance and the market and economic conditions of a country were more unstable, resulting in a crisis due to high economic uncertainty. Companies in this condition are more vulnerable, so that financial distress will increase.

H4 Financial distress moderates financial immunity to stock returns.

H5 Financial distress will be higher during the US-China trade war.

## 3 Research method

### 3.1 Data and sample

The research data used must meet the criteria: first, the companies were listed on the Indonesia Stock Exchange and the Shanghai Stock Exchange in 2016–2019. Second, Indonesia's and China's economic and government interventions data were taken from the official World Bank website. Third, all required data, including control variables, were available. Based on these criteria, the research samples were 767 observations in Indonesia and 736 observations in China. This study employed two regression models: single interaction panel data regression and multiple interaction panel data regression with a pair sample-test to determine the impact before and during the US-China trade war. The input data were balanced data, meaning that the same company was compared from time to time. The reason is that this study intends to analyze the financial immunity and vulnerability of the same company from time to time during the US-China trade war. The regression model specification test using the Chow Test resulted in the Fixed Effects Model as the best model (cross-section Chi-square probability <5%), and the Hausman test results also resulted in the Fixed Effects Model as the best model (cross-section random probability <5%). Panel data combined cross-section and time-series data making it more informative, efficient, dynamic and avoiding multicollinearity problems to minimize bias when aggregating from individuals to broader generalizations. Regression model specification of the response variable for the fixed effects model can overcome the common effects model limitations.

The dependent variable of this study is stock return, which is a percentage of the return earned by investors for the risk they bear on an investment. The greater the expected return from the investment, the higher the risk will be, so it is said that the expected return has a positive relationship with risk. The formula used to measure the stock return is as follows:

(5) $$Stock\;Return_{i,t}=\frac{stock\;price_{i,t}-stock\;price_{i,t-1}}{Stock\;price_{i,\;t-1}}$$

The independent variable of this study is financial immunity, which is measured multidimensionally with three main aspects, namely financial and market performance, economic conditions of a country, and government interventions. The moderating variable in this study is financial distress, which is measured using the Zmijewski method. The control variables used are Tobin's-Q, which is the ratio between the market capitalization value and companies' book value and firm size, which is calculated using the logarithm of total assets. Tobin's-Q is needed to see stock performance from a market perspective, while firm size is needed to naturalize the variation in companies' total assets of high total assets and low total assets on the Indonesia Stock Exchange and the Shanghai Stock Exchange. The trade war is a dummy variable. 1 is given for the period during the trade war of 2018–2019, while 0 is for the period before the trade war of 2016–2017. Meanwhile, the trade war dummy variable separates the analysis on two conditions: before and during the US-China trade war.

### 3.2 Statistical method

The model for testing the hypothesis in this study is as follows:

**Model 1:** Testing is done using panel data regression.

**H1:** Financial immunity to stock returns without being moderated by financial distress

(6) $$R_{i,t}=\alpha +ROA_{i,t}+BMV_{i,t}+PER_{i,t}+GDP_{i,t}+IF_{i,t}+CSR_{i,t}+SIZE_{i,t}+TOBINSQ_{i,t}+WAR_{t}+\varepsilon_{i,t}$$

**H4:** Financial immunity to stock returns moderated by financial distress

(7) $$R_{i,t}=\alpha +ROA_{i,t}+BMV_{i,t}+PER_{i,t}+GDP_{i,t}+IF_{i,t}+CSR_{i,t}+FD_{i,t}+FD*ROA_{i,t}+FD*BMV_{i,t}+FD*PER_{i,t}+FD*GDP_{i,t}+FD*IF_{i,t}+FD*CSR_{i,t}+SIZE_{i,t}+TOBINSQ_{i,t}+WAR_{t}+\varepsilon_{i,t}$$

Where, R_iet_ is stock return rate, ROA_i,t_ is return on assets, BMV_i,t_ is book to market value, PER_i,t_ is price earnings ratio, GDP_i,t_ is gross domestic product, IF_i,t_ is inflation, CSR_i,t_ is corporate social responsibility, FD_i,t_ is financial distress, SIZE_i,t_ is firm size, TOBINSQ_i,t_ is company's financial performance, WAR_t_ is dummy variable, 1 is for after the trade war, and 0 is for before trade war.

Model 2: Testing is done using pairwise.

**H2:** Financial immunity will be lower during the US-China trade war.

**H3:** Stock returns will be lower during the US-China trade war.

**H5:** Financial distress will be higher during the US-China trade war.

## 4 Results

Table 2 presents the test results of the effect of financial immunity on stock returns (see Table 1).

**Table 2:** Financial immunity, financial distress, and stock return (Model 1).

Variable	H1						H4					
	Indonesia			Shanghai			Indonesia			Shanghai		
	Standardized Coefficients	t	Sig.	Standardized Coefficients	t	Sig.	Standardized Coefficients	t	Sig.	Standardized Coefficients	t	Sig.
(Constant)		-2.071	0.039∗∗		-1.836	0.011∗∗		-2.278	0.023∗∗		-1.921	0.031∗∗
ROA	0.098	0.152	0.079∗	0.958	1.210	0.056∗	0.005	0.044	0.065∗	0.643	1.231	0.016∗∗
BMV	-0.870	-1.314	0.189	-0.093	-0.229	0.029∗∗	-0.149	-0.300	0.064∗	-0.071	-0.172	0.072∗
PER	0.931	1.654	0.199	0.241	0.261	0.043∗∗	0.002	0.038	0.143	0.064	0.129	0.021∗∗
GDP	1.321	2.142	0.032∗∗	0.924	1.926	0.016∗∗	1.197	2.149	0.032∗∗	0.785	1.273	0.044∗∗
IF	-1.421	-2.426	0.615	-0.879	-1.162	0.125	-1.217	-0.515	0.607	-0.890	-1.291	0.218
CSR	0.991	1.290	0.097∗	0.195	0.252	0.055∗	0.051	0.812	0.017∗∗	0.129	0.217	0.091∗
FD							-1.168	-1.323	0.086∗	0.912	1.721	0.122
ROAFD							0.001	0.009	0.048∗∗	0.731	1.284	0.025∗∗
BMVFD							-1.074	-0.608	0.043∗∗	-0.199	-0.213	0.081∗
PERFD							1.059	1.615	0.107	0.347	0.732	0.021∗∗
GDPFD							0.017	0.010	0.088∗	0.093	0.115	0.027∗∗
IFFD							-0.241	-1.163	0.025∗∗	-0.953	-1.233	0.128
CSRFD							0.923	1.086	0.078∗	0.109	0.121	0.021∗∗
Size	0.082	0.144	0.086∗	0.182	0.237	0.019∗∗	-0.032	-0.180	0.057∗	0.117	0.177	0.067∗
Tobin's Q	0.182	0.277	0.082∗	0.085	0.143	0.042∗∗	0.048	0.254	0.099∗	0.493	0.453	0.083∗
War	-1.392	-2.341	0.020∗∗	-0.932	-1.121	0.018	-1.298	-2.350	0.019∗∗	-0.978	-1.221	0.027∗∗
N			767			736			767			736
Adj. Rsquare			0.017			0.019			0.020			0.017
F-stat			4.956			4.673			5.704			4.973

∗∗∗significant at 1%.

∗∗significant at 5%.

∗significant at 10%.

**Table 1:** Research model (*Pairwise Testing*).

Model	Objection	Formula
Model 2	Pairwise Test Before and during the Trade War US-China: Stock Returns and Independent Variable	*x*R_after_ ≠ *x*R_before_ *x*ROA_after_ < *x*ROA_before_ *xBMV*_after_ < *x*BMV_before_ *x*PER_after_ < *x*PER_before_ *x*GDP_after_ < *x*GDP_before_ *x*IF_after_ < *x*IF_before_ *x*CSR_after_ < *x*CSR_before_ *x*FD_after_ > *x*FD_before_

Tests were conducted on 767 companies in Indonesia and 736 companies in China (SSE). In Table 2 for H1, financial immunity, as indicated by the ROA, BMV, PER, GDP, IF, and CSR proxies, has a different direction of relationship and significance level both between variables and between countries. Indonesia and China both have a negative direction on BMV and IF. The PER ratio has an insignificant effect on stock returns in Indonesia, while it has a significant positive effect in China. The ROA, GDP, and CSR ratios have a significant positive effect on returns in Indonesia and China. WAR shows that the dummy variable before and during the US-China trade war also has the same results, which is a significant negative effect. The control variables of size and Tobin's-Q have a significant positive effect on returns in Indonesia and China. The adjusted R-Squared value for Indonesia and China are almost similar: 0.017 for Indonesia and 0.019 for China. In the first testing without using a moderating variable, Indonesia's adjusted R-Squared was lower than China's adjusted R-Squared.

In Table 2 for H4, ROA, GDP, and CSR have a significant positive effect on returns in Indonesia. Meanwhile, PER has no significant effect on returns in Indonesia. In contrast to Indonesia, ROA, PER, GDP, and CSR in China have a significant positive effect on returns. Financial distress has a significant negative effect on Indonesia's returns, and on the contrary, China's financial distress has no significant effect on returns.

Table 3 presents the results of the matched test or Paired Samples T-Test for hypothesis testing. There are differences in results between countries and between variables caused by the US-China trade war. This table also shows the results of hypotheses 2, 3, and 5 of this study. Hypothesis 2 is accepted as financial immunity is proven to be lower during the US-China trade war in Indonesia and China. Hypothesis 3 is accepted as stock returns are lower during the US-China trade war. Hypothesis 5 is accepted as financial distress is higher during the US-China trade war in Indonesia and China.

**Table 3:** Financial immunity before and during the US-China Trade War. (Model 2: H2, H3, and H5).

Variable	WAR	Indonesia		China	
		F	Sig	F	Sig
ROA	1	7.047	0.088∗	6.521	0.018∗∗
	0				
BMV	1	10.030	0.015∗∗	8.324	0.025∗∗
	0				
PER	1	0.717	0.240	1.217	0.139
	0				
GDP	1	0.456	0.000∗∗	1.236	0.000∗∗∗
	0				
IF	1	6.500	0.000∗∗	2.124	0.000∗∗∗
	0				
CSR	1	0.799	0.091∗	1.533	0.032∗∗
	0				
FD	1	0.745	0.061∗	1.491	0.047∗∗
	0				

∗∗∗significant at 1%.

∗∗significant at 5%.

∗significant at 10%.

## 5 Discussion

### 5.1 Financial immunity and stock return

Financial immunity is indicated by the ROA, BMV, PER, GDP, IF, and CSR variables. ROA reflects the ability of company managers to manage company assets. Managers who are good in asset management (Ding et al., 2021; Grundke and Moser, 2019; C. He et al., 2021) can attract investors to buy companies' stocks. The ability of managers will have an impact on companies' financial condition, especially in making a profit. The BMV and IF variables have a negative direction, indicating that the higher the BMV and IF, the lower the resulting returns. BMV reflects companies’ value compared to their market value. A high BMV does not provide a favorable stock price difference for the companies. While IF reflects an increase in the overall price of goods in a country. Thus, domestic goods are less preferable because they seem expensive, and consequently, people prefer foreign or imported goods. This results in unattractive domestic investment for investors leading to stock prices tend to fall.

Both Indonesia and China (SSE) experienced changes after the US-China trade war. The results of this study are in line with research conducted by Grundke and Moser (2019) and He et al. (2021). The US-China trade war leads to unstable company performance and state income (Ding et al., 2021; Zaremba et al., 2021). CSR is also a corporate strategy in forming a positive brand image as a company that cares about the environment and obeys government regulations (Ajina, 2019; Alsaadi et al., 2017; Boubaker et al., 2020). Socially responsible companies that have suitable investments attract more investors. The war variable is used to see conditions before and during the US-China trade war. A significant negative result indicates that financial immunity and returns in Indonesia and China diminish during the US-China trade war. Companies with financial immunity will be more immune to risk, so investors are interested in investing in the companies because the companies' prospects are good. The hope is that investors get high returns because of the tendency for the companies’ stock prices and stock value to increase. This premise is supported by Dogah (2021) that reveals the effect of trade policy uncertainty on economic policy uncertainty in the ASEAN region. This domino effect is due to the trade war involving the two countries with the largest global trade so that the policies issued by the two countries have global effects and a domino effect (Li et al., 2020). In line with He et al. (2021), this present study proves that the US-China trade war significantly reduces financial immunity and stock returns in China and Indonesia.

### 5.2 Financial immunity, financial distress, and stock return

Financial distress has a significant negative effect on Indonesia's returns, and on the contrary, China's financial distress has no significant effect on returns. This difference in results is possible because the financial distress experienced by different country levels gives different results. Even in an unfavorable condition, it turns out that companies in China can still survive in overcoming financial distress. As one of the countries with a strong economy, companies in China can still pay their short- and long-term obligations during the US-China trade war. Meanwhile, financial distress in Indonesia weakens financial immunity so that the resulting return will decrease. Different results are found in China in which the financial distress has not reached the limit that can reduce financial immunity; thus, the immunity level is still acceptable, which subsequently makes stock returns is still controllable.

These results are supported by Chang (2021) and Yu and Huang (2021), which recommend that the impact of economic policy uncertainty on stock market volatility is highly dependent on each country's characteristics. The impact of the US-China trade war on the Indonesia Stock Exchange and the Shanghai Stock Exchange has increased the uncertainty of government policies for business regulations, primarily on the determination of product prices and costs in international trade traffic. There was a significant reaction from stock market participants on policies during the US-China trade war, which greatly influenced investment decisions. Trade policy uncertainty impacts a volatile economy, causing investors to behave more conservatively. Investors save their assets through safer investment allocations to anticipate potential ongoing risks. Therefore, during the US-China trade war, trade policy uncertainty has caused economic policy uncertainty, thus triggering systemic risks in ASEAN markets, including Indonesia. Dogah (2021) reveals that bilateral relations and trade affect policy uncertainty. This is understandable considering that the US and China are essential players in the global economy, owners of capitalization, and investing large amounts of money in many countries.

China's trade policy uncertainty contributes to the most substantial contagion effect on ASEAN markets, including Indonesia. Therefore, a solid bilateral trade relationship between China and Indonesia from the supply side is a critical determinant of corporate financial immunity to the global policy uncertainty challenge. This relationship is further strengthened by the high trade dependence and transnational investment associations between China and ASEAN (Yang et al., 2021). At first, the impact of the US policy uncertainty was very significant, but its impact on the ASEAN market gradually began to diminish (Dogah, 2021). Controlling the uncertainty is very strategic because it will likely weaken companies' financial immunity. Gerrans, Faff and Hartnett (2015) examine that some investors shift risky assets in their portfolios when the economic crisis hits the world. This value creation is backed up by government policies in each country to retain a conducive business climate and acceleration to maintain companies' economic value.

## 6 Conclusions, suggestions, and implications

Financial immunity from the impact of the US-China trade war has never been studied, especially in developing countries like Indonesia. The multi-country analysis in this study reveals that financial immunity has a direct impact on the US-China and an indirect impact on Indonesia as the largest export destination country. The economic impact felt by Indonesia is very significant as Indonesia's trade balance is in a deficit position compared to China. This becomes more serious because Indonesia's largest non-oil and gas import market is China. Meanwhile, Indonesia is not overly dependent on imports from the US.

The test results of model 1 show that financial immunity has a positive effect on returns in Indonesia and China. While the moderating variable in the two countries shows different results. Indonesia's financial distress has a significant negative effect on returns, and on the contrary, China's financial distress has no significant effect on returns. The test results of model 2 reveal that both Indonesia and China experience changes in financial immunity, stock returns, and financial distress after the US-China trade war. This reflects that the US-China trade war has significantly affected both macroeconomic and firm performance.

For future research, it is recommended to develop further from the policy aspect to see which policy uncertainty fundamentally contributes to the most significant impact on the systemic risk of capital markets in China and Indonesia during the US-China trade war. A previous study by Dogah (2021) revealed that the significant effect of risk has shifted from developed to developing countries, and the most significant influence was triggered by uncertainty in economic policies from China to countries in ASEAN, while the impact from the US gradually began to diminish. Although the US initially triggered the trade war, China is currently the most influential contributor to regional systemic risks related to financial immunity.

This present study can have implications for companies in developing and developed countries and policy-making bodies in a country. This suggests structuring China's economic and trade policies in emerging markets because trade connectivity is the main source of risk contagion. While future concerns will be on counter-macroprudential policy-making that hedges against market volatility to maintain financial immunity. The strategy taken can be done through exposure to collaboration in international trade policies because it is presumed that developing countries cause uncertainty shocks. Therefore, to improve financial immunity after the US-China trade war, it is necessary to consider reforming regional capital market investment connectivity policies in dealing with the ASEAN and Chinese economic swells.

The multi-country analysis performed in this study does not involve all developing countries, especially ASEAN countries other than Indonesia. Therefore, the impact of the US-China trade war on emerging markets could not be fully revealed for each ASEAN country. Moreover, it is necessary to consider dimensions that have the potential to affect financial immunity and vulnerability in times of crisis, namely during the US-China trade war.

## Declarations

### Author contribution statement

Liliana Inggrit Wijaya and Zunairoh Zunairoh: Conceived and designed the experiments; Performed the experiments; Analyzed and interpreted the data; Contributed reagents, materials, analysis tools or data; Wrote the paper.

Rizky Eriandani: Analyzed and interpreted the data; Contributed reagents, materials, analysis tools or data.

I Made Narsa: Contributed reagents, materials, analysis tools or data.

### Funding statement

This research did not receive any specific grant from funding agencies in the public, commercial, or not-for-profit sectors.

### Data availability statement

Data will be made available on request.

### Declaration of interests statement

The authors declare no conflict of interest.

### Additional information

No additional information is available for this paper.
